# Pathologic Complete Response Rates in Early-Stage Human Epidermal Growth Factor 2 (HER2)-Positive Breast Cancer Treated With Neoadjuvant Chemotherapy and Anti-HER2 Therapy: A Real-World Experience

**DOI:** 10.7759/cureus.99779

**Published:** 2025-12-21

**Authors:** Rania Mokfi, Farah Boutaagount, Meryem Maskrout, Soundous Bennour, Chaymae Senoussi, Lamia Nadif, Naoufal Berrada, Latifa Lamine, Ichrak Bouhou, Sarah Naciri, Ghizlane Rais

**Affiliations:** 1 Department of Medical Oncology, Faculty of Medicine and Pharmacy of Agadir, Ibn Zohr University, University Hospital Center Souss-Massa, Agadir, MAR; 2 Department of Medical Oncology, Sidi Mohamed Ben Abdellah National Institute of Oncology, Rabat, MAR

**Keywords:** breast, chemotherapy, her2, pertuzumab, trastuzumab

## Abstract

Background

Achieving a pathological complete response (pCR) is a major goal of neoadjuvant therapy for early-stage human epidermal growth factor 2 (HER2)-positive breast cancer. The introduction of anti-HER2 agents into chemotherapy has significantly increased pCR rates. Trastuzumab, the first humanized monoclonal antibody targeting the extracellular domain of HER2, improves both pCR and event-free survival when added to standard regimens. Pertuzumab, which binds to domain II of HER2 and inhibits HER2-HER3 dimerization, provides complementary activity. Despite these advances, additional biomarkers predicting pCR are still being explored. This study aimed to assess pCR rates and identify clinicopathological factors associated with improved response and survival.

Methodology

Our retrospective study included 140 patients with early HER2-positive breast cancer who received neoadjuvant chemotherapy (NAC) at the Regional Oncology Center in Agadir between January 2018 and December 2022. Logistic regression was applied to evaluate the impact of clinico-pathological variables on pCR, and a p-value below 0.05 was considered statistically significant. Kaplan-Meier curves were used to assess progression-free survival (PFS) and overall survival (OS). Survival comparisons were performed using the log-rank test. Multivariate Cox regression models were constructed to evaluate the impact of different covariates on pCR.

Results

The median age of the cohort was 49.5 ± 10.2 years. Among the 140 patients, 58 (41.4%) were diagnosed at stage II and 82 (58.6%) at stage III. Lymph node involvement was found in 74 cases (52.9%), and hormone receptor (HR) positivity was noted in 77 patients (55%). Following curative surgery, pCR was achieved in 72 patients (51.4%). Multivariate analysis identified age <50 years (p = 0.043), lymph node involvement (p < 0.001), nuclear grade 3 (p < 0.001), and Ki67 ≥35% (p < 0.001) as independent predictors of pCR. Overall, the three-year PFS and the three-year OS rates were 95.5% (95% CI: 92.0-99.1%) and 98.4% (95% CI: 96.2-100%), respectively. Patients who achieved pCR demonstrated significantly longer PFS compared with those who did not (three-year PFS: 98.6% vs. 92.3%, p = 0.027). The most frequently reported adverse events (AEs) were fatigue in 92 cases (65.7%), anemia in 92 cases (65.7%), and nausea/vomiting in 77 patients (55%). The most common grade 3-4 AEs were fatigue in 22 patients (15.7%), nausea/vomiting in 13 patients (9.3%), and anemia in 11 patients (7.8%). Cardiac toxicity occurred in 10 patients (7.1%).

Conclusions

Anti-HER2 therapies combined with NAC improved pCR rates while maintaining a manageable safety profile. Clinicopathological factors such as tumor grade, nodal status, HR status, and Ki67 also influenced pCR. However, emerging predictive biomarkers are currently under investigation to further optimize escalation and de-escalation treatment strategies. Future studies on larger cohorts incorporating these additional biomarkers are needed to enhance predictive models and guide personalized therapy.

## Introduction

Pathological complete response (pCR) is defined as the absence of residual invasive carcinoma in the resected breast and axillary tissue following neoadjuvant chemotherapy (NAC). Achieving pCR is a central goal of neoadjuvant therapy in early-stage human epidermal growth factor 2 (HER2)-positive breast cancer [1], as it is associated with improved long-term outcomes, including progression-free survival (PFS) and overall survival (OS), and can facilitate tumor downstaging [2,3]. To increase pCR rates, targeted anti-HER2 therapies have been combined with NAC. Trastuzumab, a humanized monoclonal IgG1 antibody targeting the extracellular domain of HER2, has achieved pCR rates of 25-46% [4,5]. Pertuzumab, a second-generation anti-HER2 agent, not only induces antibody-dependent cellular cytotoxicity but also inhibits HER2 signaling by preventing its heterodimerization with partner receptors [3]. The combination of trastuzumab and pertuzumab has further improved pCR rates to 49-66% [5], establishing dual anti-HER2 therapy with chemotherapy as the standard neoadjuvant regimen [6]. Despite these advances, identifying novel predictive biomarkers and determining which patients are most likely to benefit from neoadjuvant therapy remain important areas of ongoing research [7]. To the best of our knowledge, only a few studies have been reported in the Moroccan population. The objective of our study is to evaluate the pCR rate following neoadjuvant therapy in a real-world cohort of patients with HER2-positive early breast cancer and to identify clinicopathological factors associated with a higher probability of achieving pCR and longer survival.

## Materials and methods

Patients

This retrospective study included 140 patients with early-stage HER2-positive breast cancer who received NAC at the Regional Oncology Center in Agadir between January 2018 and December 2022.

**Figure 1:**
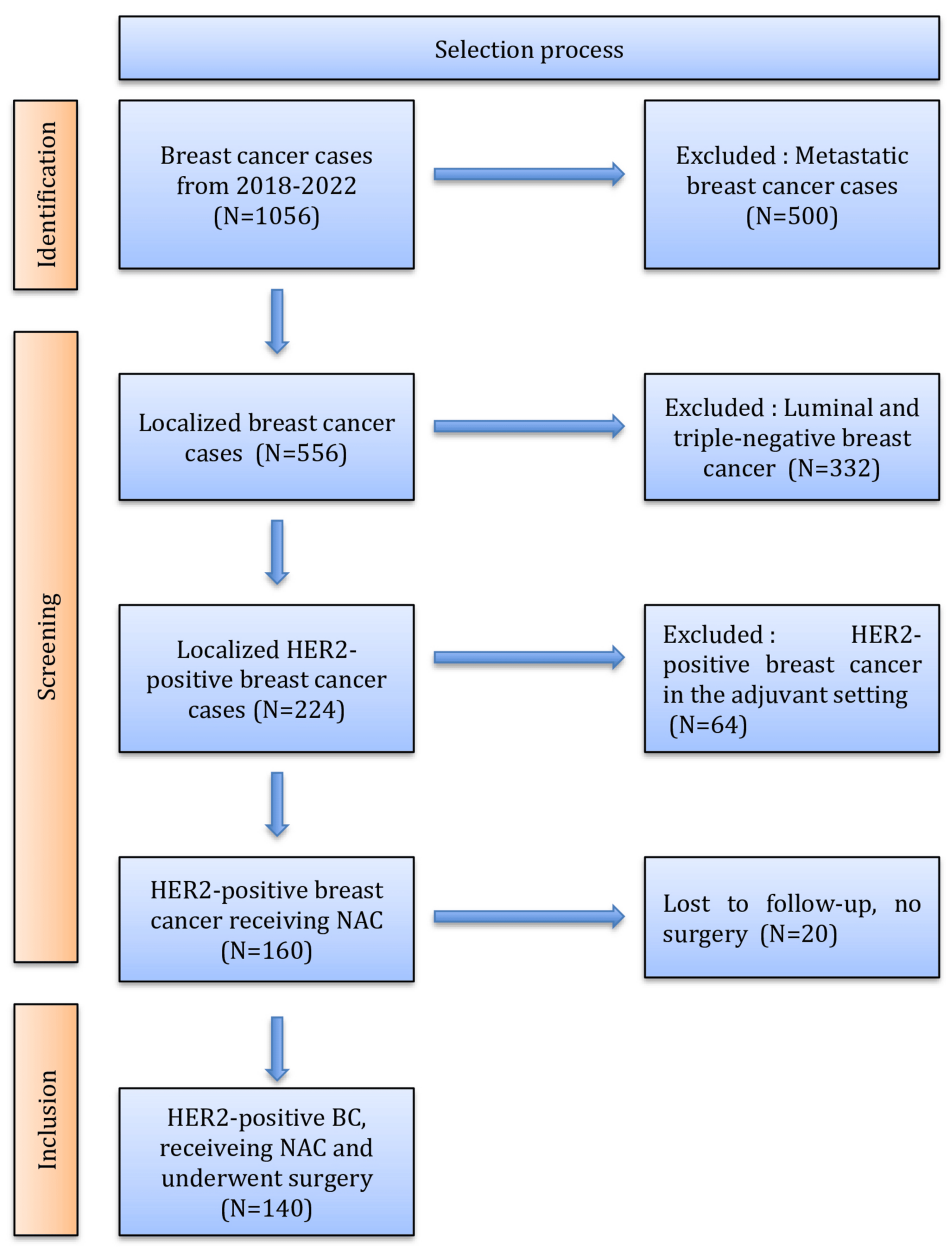
Selection process HER2: Human epidermal growth factor receptor 2; NAC: Neoadjuvant chemotherapy; BC: Breast cancer

Patients were enrolled only if they had completed NAC, followed by surgery. The inclusion criteria for NAC administration were: Confirmed invasive breast cancer, clinical stage T2-T4 and/or lymph node involvement. Patients’ overall health status and ability to tolerate chemotherapy were also considered. The study selection process is summarized in the flowchart presented in Figure 1.

The study was approved by the Local Ethics Committee of CHU Souss Massa, University Ibn Zohr, Agadir (approval no. 01_12_2024, 01 December 2024) and conducted in accordance with the Declaration of Helsinki. Written informed consent was obtained from all participants.

Data collection

Clinical and histopathological information was obtained through retrospective review of electronic patient records. The collected variables included demographic and clinical characteristics (age, medical history, menopausal status, CA15-3 level), tumor-related parameters (tumor size, nodal status, disease stage, histopathological features such as tumor grade, molecular subtype, and Ki-67 index), treatment-related information (neoadjuvant chemotherapy regimens, neoadjuvant targeted therapy regimens, adverse events (AEs) associated with neoadjuvant therapy, surgical interventions, and adjuvant treatments including radiotherapy and endocrine therapy).

Pathological analysis

Histological parameters were documented from anatomopathological reports, including histological type, tumor grade as defined by the SBR system (Scarff-Bloom Richardson), estrogen receptor (ER) status, progesterone receptor (PR) status, and Ki-67 value.

Molecular profiling was conducted using monoclonal mouse antibodies (for ER: ID5, 1:50 dilution; Dako, Glostrup, Denmark), (for PR: PgR636, 1:100 dilution; Dako) and HER-2 protein (CB11, 1:100 dilution; NeoMarker, Fremont, USA). Molecular subtypes were defined according to the 2013 Saint Gallen consensus.

Hormone receptor (HR) positivity was defined as ≥1% expression of either ER or PR. HR-negative status was defined as the complete absence of ER and PR expression in tumor cells. HER2 status was evaluated by immunohistochemistry (IHC) using the HercepTest. HER2 positivity was defined as either a score of 3+ on IHC or a score of 2+ on IHC confirmed by fluorescence in situ hybridization (FISH). Proliferative activity was assessed by Ki-67 expression, evaluated through automated quantitative image analysis following immunostaining with a monoclonal antibody (MIB1, dilution 1:400; Dako, Denmark). Digitized tissue sections were acquired at high resolution (40× objective), and computer-assisted algorithms were applied to detect and quantify Ki-67-positive nuclei.

Treatment protocols

All patients included in this study received NAC in combination with dual anti-HER2 therapy, in accordance with current clinical guidelines. NAC consisted of three to four cycles of anthracycline-based regimens, either AC60 (doxorubicin 60 mg/m² and cyclophosphamide 600 mg/m²) or EC100 (epirubicin 100 mg/m² and cyclophosphamide 500 mg/m²), administered every three weeks, followed by taxane-based therapy. The taxane regimen included either weekly paclitaxel (80 mg/m²) or triweekly docetaxel (100 mg/m²).

Anti-HER2 targeted therapies were administered in combination with taxanes. Patients received either dual HER2 blockade with pertuzumab (loading dose 840 mg intravenously, followed by 420 mg every three weeks) combined with trastuzumab (600 mg subcutaneously every three weeks), or trastuzumab monotherapy (600 mg subcutaneously every three weeks). The selection between dual HER2 blockade and trastuzumab monotherapy was dictated by the accessibility of pertuzumab, which was not available at the center prior to 2021.

All patients underwent either mastectomy or breast-conserving surgery with axillary lymph node dissection. The choice of surgical approach was determined during a multidisciplinary team meeting, taking into account the initial disease stage, the feasibility of achieving optimal cosmetic outcomes, and patient preference.

Adjuvant therapy was determined based on histopathological response, initial nodal status, and the neoadjuvant anti-HER2 regimen. Patients received either dual HER2 blockade with pertuzumab and trastuzumab or trastuzumab monotherapy. Trastuzumab-emtansine (T-DM1) was not available in Moroccan centers at the time of the study. Dual HER2 therapy was continued in patients with residual nodal disease and/or those who did not achieve a pathological complete response.

Evaluation of pathological response

Pathological response was assessed in surgical breast specimens. Total pathological complete response (pCR) was defined as the complete absence of invasive disease in both the breast and axillary lymph nodes (ypT0/ypN0). pCR evaluation was conducted using the Sataloff and Chevalier classification systems. At the time of the study, the Residual Cancer Burden (RCB) classification had not been implemented in Moroccan centers. 

Toxicity assessment

AEs were categorized according to the Common Terminology Criteria for Adverse Events (CTCAE) version 5.0. Cardiotoxicity was defined as a reduction in left ventricular ejection fraction (LVEF) of ≥ 10% from baseline, or an LVEF falling below 50%, and/or the occurrence of Class III or IV congestive heart failure according to the New York Heart Association classification.

Statistical analysis

Quantitative variables were expressed as mean ± standard deviation (SD), whereas qualitative variables were presented as frequencies and percentages. The distribution of continuous variables was assessed using the Shapiro-Wilk test. Comparisons of numerical variables were performed using the Mann-Whitney U test, while categorical variables were analyzed using the chi-square or Fisher’s exact test, as appropriate. Multivariate logistic regression analysis was conducted to evaluate the impact of various parameters on pCR, with results expressed as odds ratios (ORs) and 95% confidence intervals (CIs). 

Receiver operating characteristic (ROC) curve analysis and the area under the curve (AUC) were employed to determine the optimal Ki-67 cutoff value, defined as the point corresponding to maximal sensitivity and minimal 1-specificity.

PFS was defined as the interval from the date of surgery to the first occurrence of local or distant recurrence. OS was calculated from the date of surgery to death or the end of follow-up. Survival curves were estimated using the Kaplan-Meier method, and differences between groups were assessed with the log-rank test. Multivariate Cox proportional hazards regression models were constructed to evaluate the impact of covariates on pCR and PFS. A two-sided P-value of <0.05 was considered statistically significant. All analyses were performed using Jamovi software (https://www.jamovi.org).

## Results

Clinical and histological characteristics

A total of 140 patients were included, with a median age of 49.5 ± 10.2 years. At diagnosis, 75 patients (53.6%) were premenopausal and 65 (46.4%) were postmenopausal. Tumors >5 cm were observed in 70 patients (50%). Comorbidities included diabetes mellitus in 7.9% (11), arterial hypertension in 2.1% (3), and a family history of breast cancer in 12.1% (7). ECOG (Eastern Cooperative Oncology group) performance status was 1 in 135 cases (96.4%) and 2 in five cases (53.6%). Lymph node involvement was found 74 patients (52.9%), and the mean CA 15-3 level was 10.8 ± 9.3 U/mL. Stage at diagnosis was II in 58 patients (41.4%) and III in 82 patients (58.6%) (Table 1).

**Table 1:** Patient characteristics SD: Standard deviation; ECOG: Eastern Cooperative Oncology Group; SBR: Scarff-Bloom-Richardson; HR: Hormone receptor; NAC: Neoadjuvant chemotherapy

Variables	Value (%)
Age (Mean ± SD)	49.5± 10.2
Menopausal status	
Premenopausal	75 (53.6%)
Postmenopausal	65 (46.4%)
ECOG Performance Status Scale	
1	135 (96.4%)
2	5 (3.6%)
Stage	
II	58 (41.4%)
III	82 (58.6%)
Tumor size (cm)	
<2 cm	8 (5.7%)
2-5cm	62 (44.3%)
>5 cm	70 (50%)
Lymph node status	
Positive	74 (52.9%)
Negative	66 (47.1%)
Histological type	
Invasive ductal carcinoma	125 (89.3%)
Invasive lobular carcinoma	15 (10.7%)
Histological grade (SBR)	
Grade 2	73 (52.1%)
Grade 3	67 (47.9%)
Vascular invasion	
Positive	71 (50.7%)
Negative	69 (49.3%)
HR status	
Positive	77 (55%)
Negative	63 (45%)
Ki‐67 index	
≥35%	127 (90.7%)
<35%	13 (9.3%)
Response to NAC	
pCR	72 (51.4%)
No pCR	68 (48.6%)
Treatment regimens	
Anthracycline + cyclophosphamide followed by Taxanes + Trastuzumab	97 (69.3%)
Anthracycline + cyclophosphamide followed by Taxanes + Trastuzumab and Pertuzumab	43 (30.7%)
Breast surgery	
Mastectomy + Axillary lymphadenectomy	114 (81.4%)
Breast conserving + Axillary lymphadenectomy	26 (18.6%)

Invasive ductal carcinoma was the predominant histological subtype, observed in 125 patients (89.3%), whereas invasive lobular carcinoma was found in 15 patients (10.7%). Histological grade 2 was most frequent, observed in 52.1% of patients (n = 73), while grade 3 was identified in 47.9% (n = 67). Vascular invasion was present in 71 patients (50.7%). HR positivity was noted in 77 patients, representing 55% of the cohort.

Based on the ROC curve analysis, the AUC was 0.771 (95% CI: 0.668-0.882), indicating good discriminative ability. A cutoff value of 35% was established for Ki-67 expression. Among the study population, 127 patients (90.7%) had a Ki-67 level ≥35%, whereas 13 patients (9.3%) had a Ki-67 level <35% (Table 1).

Neoadjuvant therapy consisted of chemotherapy combined with dual anti-HER2 blockade in 43 patients (30.7%) and trastuzumab monotherapy in 97 patients (69.3%). Following NAC, 82 patients (58.6%) achieved a complete clinical response, 46 patients (32.9%) demonstrated a partial clinical response, and 12 patients (8.6%) had stable disease.

Surgery was performed following completion of NAC with a median interval of 24.7 ± 3.68 days. pCR was achieved in 72 patients (51.4%). Adjuvant therapy consisted of trastuzumab monotherapy in 110 patients (78.5%) and dual HER2-targeted therapy in 30 patients (21.5%), with a median duration of 12 ± 1.8 months. 

Long-term outcomes: progression-free survival and overall survival

**Figure 2:**
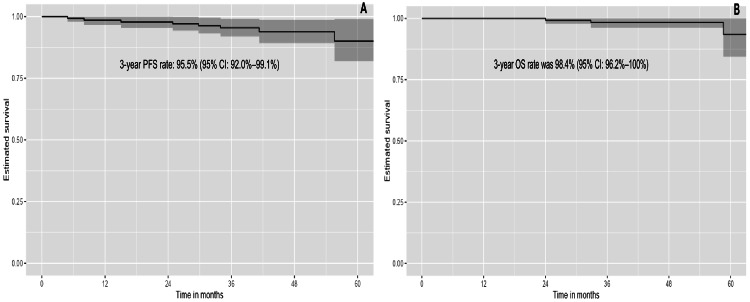
Progression-free survival (A) and overall survival (B) for the entire population PFS: Progression-free survival; OS: Overall survival; CI: Confidence Intervals

The median follow-up was 38.4 months. Median PFS and OS were not reached at the end of the follow-up period. The estimated three-year PFS rate was 95.5% (95% CI: 92.0%-99.1%), and the three-year OS rate was 98.4% (95% CI: 96.2%-100%) (Figures 2A, 2B).

Association between clinico-pathological variables and pCR

Univariate analysis demonstrated a significant association between pCR and age <50 years (p = 0.004), tumor size <5 cm (p = 0.005), lymphatic invasion (p = 0.001), nuclear grade 3 (p = 0.003), vascular invasion (p = 0.001), and Ki-67 index ≥35% (p < 0.001). No significant association was observed between pCR and family history of cancer, menopausal status, tumor location, hormone receptor status, or treatment regimen (Table 2).

**Table 2:** Univariate and multivariate analysis of predictive factors of pathological complete response OR: Odds ratio; CI: Confidence interval; HR: hormone receptor

Variable	Univariate analysis		Multivariate analysis	
	OR (95% CI)	P-Value	OR (95% CI)	P-Value
Age (< 50 vs ≥50)	0.886 (0.160-0.980)	0.004	0.915 (0.149-0.986)	0.043
Menopausal status (Menopausal vs premenopausal)	0.990 (0.977-3.170)	0.006	⎯	⎯
Tumor location (Right vs Left)	0.590 (0.446-1.020)	0.612	⎯	⎯
Tumor size (<5cm vs ≥ 5 cm)	0.908 (0.446-0.997)	0.005	1.780 (0.510-3.494)	0.994
Lymph node status (Positive vs negative)	0.847 (0.260-0.947)	0.001	0.870 (0.377-0.963)	<0.001
Histological type (Ductal vs Lobular)	0.852 (0.391-0,913)	<0.001	0.842 (0.611-1.188)	0.052
Nuclear grade (3 vs 2)	1.197 (1.020-5.877)	0.003	1.270 (1.137-7.776)	<0.001
Vascular invasion (positive vs negative)	0.520 (0.230-0.954)	0.001	0.440 (0.258-3.468)	0.994
Ki67 (≥ 35% vs <35%)	1.260 (1.015-3.770)	<0.001	1.520 (1.010-3.689)	<0.001
HR status (positive vs negative)	0.360 (0.302-1.447)	0.070	⎯	⎯
Treatment regimens (Pertuzumab + Trastuzumab vs Trastuzumab monotherapy)	2.020 (1.809-5.069)	0.388	⎯	⎯

Multivariate analysis identified age <50 years (p = 0.043), lymph node involvement (p < 0.001), nuclear grade 3 (p < 0.001), and Ki-67 ≥35% (p < 0.001) as independent predictive factors for achieving pCR (Table 2).

Survival outcomes (PFS and OS) based on pCR status

**Figure 3:**
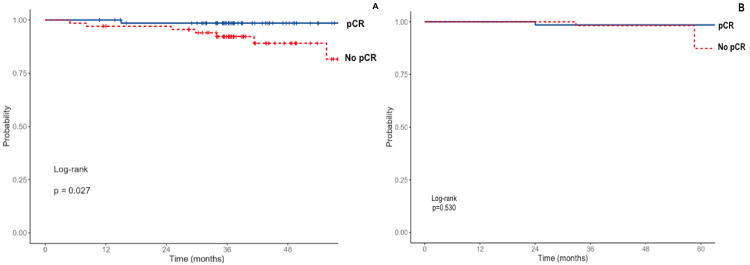
Kaplan-Meier Survival curves illustrating progression-free survival (A) and overall survival (B) according to pCR status pCR: Pathological complete response

Patients who achieved pCR had significantly longer PFS (three-year PFS: 98.6% vs. 92.3%; p = 0.027), whereas no significant difference was observed in overall survival between patients with and without pCR (three-year OS: 98.6% vs. 98.3%; p = 0.530) (Figures 3A, 3B).

Safety

The most frequently observed AEs were fatigue and anemia, each affecting 92 patients and representing 65.7% of the study population. Nausea and vomiting occurred in 77 patients (55%), hair loss and neutropenia in 65 patients (46.4%), diarrhea in 44 patients (31.4%), peripheral neuropathy in 38 patients (27.1%), decreased cardiac function and elevated transaminases in 10 patients (7.1%), and rash in eight patients (5.7%). Most of these toxicities were grade 1-2. The most common grade 3-4 AEs were fatigue in 22 patients (15.7%), nausea and vomiting in 13 (9.3%), and anemia in 11 (7.8%). Cardiac toxicity was observed in 10 patients (7.1%), leading to temporary interruption of anti-HER2 therapy for a median duration of 42 ± 12 days. Permanent treatment discontinuation occurred in one patient (0.7%) due to heart failure. Toxicities are presented in Table 3.

**Table 3:** Treatment-related toxicities

Toxicity	All grade	Grade≥3
Fatigue	92 (65.7%)	22 (15.7%)
Anemia	92 (65.7%)	11 (7.8%)
Nausea and/or Vomiting	77 (55%)	13 (9.3%)
Hair loss	65 (46.4%)	0 (0%)
Neutropenia	65 (46.4%)	9 (6.4%)
Diarrhea	44 (31.4%)	9 (6.4%)
Peripheral neuropathy	38 (27.1%)	9 (6.4%)
Decrease in cardiac function	10 (7.1%)	1 (0.7%)
Elevated transaminase	10 (7.1%)	2 (1.4%)
Rash	8 (5.7%)	0 (0%)

## Discussion

Human epidermal growth factor receptor 2 (ERBB2, HER2 or HER2/neu) is a molecular target consisting of a transmembrane tyrosine kinase like receptor. This epidermal growth factor is overexpressed or amplified in approximately 20% of breast cancers [8]. Moreover, HER2-positive status is an important predictive marker of response to anti-HER2 targeted therapy such as the monoclonal antibodies trastuzumab and pertuzumab [9]. Trastuzumab is a monoclonal antibody directed against domain IV of the extracellular region of the HER2 protein [4]. Trastuzumab inhibits signaling, angiogenesis, and proliferation in HER2-overexpressing breast cancer cells. Pertuzumab is a monoclonal antibody that binds domain II of the extracellular region of the HER2 protein. This antibody was designed to disrupt the ligand-dependent heterodimerization of HER2 with other members of the HER family, most notably HER3 [10].

The incorporation of anti-HER2 therapy into NAC has consistently been associated with improved pathological complete response (pCR) rates, as demonstrated in both real-world studies and randomized clinical trials. Early evidence by Buzdar et al. was among the first to evaluate trastuzumab in the neoadjuvant setting for HER2-positive early breast cancer, reporting a pCR rate of 66% [11]. Subsequent phase II trials further demonstrated the added benefit of dual HER2 blockade. Notably, the NeoSphere trial randomized 417 patients with HER2-positive breast cancer to four treatment arms, docetaxel plus trastuzumab, docetaxel plus trastuzumab and pertuzumab (THP), trastuzumab plus pertuzumab (HP), and docetaxel plus pertuzumab. The THP arm achieved the highest pCR rate at 46%, highlighting the synergistic effect of dual HER2 inhibition when combined with chemotherapy [12]. Similarly, the TRYPHAENA trial, which included 225 patients with early-stage HER2-positive disease, reported pCR rates of up to 66% in the dual blockade arm [13]. These findings collectively establish dual HER2 blockade as a cornerstone of contemporary neoadjuvant therapy in this patient population. In contrast, lower pCR rates have been observed in other studies. Analyses from the German PRAEGNANT network and other multicenter cohorts reported slightly lower pCR rates of 52.8% and 46.8%, respectively [3], which are comparable to the 51.4% observed in our cohort. This discrepancy may be explained by the fact that the majority of patients in our study received trastuzumab monotherapy rather than a dual HER2-targeted regimen. Additionally, variations in clinicopathological characteristics across studies likely contribute to the observed differences in pCR rates.

Beyond therapeutic regimens, tumor-related factors have also been investigated as predictors of pCR. Tumor size, in particular, has been shown to influence response. Bilici et al. reported that lower T stage (T1 vs. T2-T4) was significantly associated with higher pCR rates (49.7% vs. 29.8%, p < 0.001) among 1,528 HER2-positive patients receiving neoadjuvant chemotherapy with trastuzumab, with or without pertuzumab [14]. Similarly, Jin et al. found that smaller tumors were more likely to achieve pCR, with rates of 23.6% T1 tumors versus 10.3% for T4 tumors [15]. This association may reflect reduced intratumoral heterogeneity, better drug penetration, higher proliferative activity, and more effective immune-mediated mechanisms in smaller tumors. However, our study didn’t find a significative association between Tumor size and pCR rate. This could be due to the limited sample size, the predominance of trastuzumab monotherapy and the influence of other clinicopathological characteristics.

In addition to tumor size, histological grade is another important factor influencing pCR. Literature data consistently demonstrate a correlation between higher tumor grade and increased pCR rates in early HER2-positive breast cancer. In a study by Rodriguez et al., tumor grade was significantly associated with pCR among 467 patients, with rates of 13% for grades 1-2 versus 24% for grade 3 tumors [16]. Similarly, Bilici et al. reported higher pCR rates in grade 3 tumors compared with grade 2 (45.5% vs. 28.7%, p < 0.001) among 1,528 HER2-positive patients receiving neoadjuvant therapy [14]. In fact, higher grade has been consistently associated with increased sensitivity to NAC due to higher proliferation rates and reduced differentiation.

Another factor potentially influencing pCR is tumor proliferation, commonly evaluated using the Ki-67 index. Tumors with higher Ki-67 levels are generally more likely to achieve pCR following NAC with dual anti-HER2 therapy, likely reflecting the increased chemosensitivity of rapidly dividing cells [17]. Our study confirms this association, with a pCR rate of 85.5% in patients with Ki-67 ≥35% (p < 0.001). However, the optimal Ki-67 cutoff for predicting pCR remains debated, with reported cutoff values ranging from 5% to 60% [18]. For instance, a Hungarian real-world study of 82 HER2-positive patients identified Ki-67 >60% as predictive of higher pCR rates [19], while Resende et al. in a cohort of 310 patients with early HER2-positive breast cancer found Ki-67 ≥50% to be a significant predictor [20]. Other studies have reported lower values: Yang et al. observed higher pCR rates with Ki-67 >30% in 353 patients [1], González et al. identified Ki-67 >20% as an independent predictor of pCR [6], and Bilici et al. reported increased pCR rates in tumors with Ki-67 >14% [14]. The variation in Ki-67 cutoffs between studies may be explained by differences in patient characteristics, treatment regimens, laboratory methods, and statistical approaches.

Lymph node involvement is a well-recognized prognostic factor in HER2-positive breast cancer; however, its predictive value for achieving pCR remains uncertain. While studies by Boér et al. and Hamy-Petit et al. found no significant association between nodal status and pCR [19,21], Chou et al., in a pooled analysis of 1,047 patients, demonstrated that clinical N0 and N1 disease were associated with higher pCR rates compared with the N2 stage [22]. Consistently, in our series, nodal status was significantly associated with response to NAC (OR 1.17; 95% CI 0.883-2.330; p<0.001). These findings indicate that limited nodal involvement (N0-N1) may reflect tumors with greater chemosensitivity, whereas more advanced nodal disease (N2-N3) could correspond to biologically more resistant tumors.

Moreover, the chemotherapy regimens may impact pCR rates. Across several trials, pCR rates were comparable between anthracycline-containing and anthracycline-free regimens combined with dual HER2 blockade. TRYPHAENA and TRAIN-2 both showed similar outcomes with or without anthracyclines, while NEOPERSUR even reported higher pCR rates with non-anthracycline regimens [6,13,23]. These findings highlight the potential for therapeutic escalation or de-escalation in certain HER2-positive tumors. Several predictive markers of response to anthracyclines have been identified. In the TRAIN-2 trial, benefit from anthracycline-based NAC was observed in HR-negative tumors with high Ki-67 [23]. Additionally, concurrent TOP2A amplification in HER2-positive tumors may enhance sensitivity to anthracyclines, suggesting its potential as a predictive biomarker [24]. Bayraktar et al. demonstrated that patients with larger tumors or positive lymph nodes, including those with inflammatory tumors or N2-N3 disease at diagnosis, derive greater benefit from anthracycline-containing regimens [25]. Collectively, these data suggest that tumor biology, proliferative activity, and disease burden can guide personalized decisions regarding the use of anthracyclines in the neoadjuvant setting.

Recently, new biological markers predictive of response to NAC have been reported in several studies. In particular, PIK3CA (phosphatidylinositol 3-kinases) mutations have emerged as a relevant factor, being associated with decreased pCR rates and less favorable outcomes under anti-HER2 therapy. A prospective evaluation of 737 patients from the GeparSixto and GeparQuinto trials revealed that PIK3CA mutations were predictive of resistance to combined anti-HER2 therapy and chemotherapy. Within this cohort, the pCR rate was lower in mutated tumors (6.5%) compared with the wild-type group (30.8%; p=0.005) [26].

Alterations in PTEN (Phosphatase And Tensin Homolog) expression have also been associated with treatment response. Low PTEN expression may serve as a biomarker to identify patients whose tumors are resistant to trastuzumab but remain sensitive to Lapatinib [27-29]. Moreover, higher levels of TILs (tumor-infiltrating lymphocytes) have been shown to enhance the response to trastuzumab and chemotherapy. In the GeparQuattro trial, every 10% increase in TILs was associated with a higher likelihood of achieving pCR following neadjuvant trastuzumab and NAC (adjusted odds ratio 1.14; 95% CI 1.01- 1.29; p = 0.037) [30]. These findings underscore the complexity of predicting pCR in HER2-positive breast cancer and highlight the necessity of developing comprehensive predictive models that integrate both established clinicopathological variables and emerging molecular biomarkers.

pCR is widely recognized as a surrogate marker of survival in breast cancer treated with neoadjuvant therapy [31]. Stjepanovic et al. demonstrated, in a cohort of 161 patients with breast cancer, a significant improvement in three-year PFS among patients who achieved a pCR compared with those with residual disease (97.1% vs. 89.3%, P = 0.0011) [17]. Consistent results were observed in major trials such as NeoSphere, TRYPHAENA, and KRISTINE, where patients with pCR achieved superior three-year progression-free survival compared with those without pCR [6,13,32]. Real-world evidence further confirmed this association, reporting favorable survival outcomes among patients achieving pCR [2]. Similarly, in our cohort, three-year PFS was significantly higher in the pCR group, whereas OS did not differ significantly, likely due to the limited sample size and relatively short follow-up.

Across pivotal trials, the most common AEs associated with neoadjuvant anti-HER2 therapy were chemotherapy-related toxicities, predominantly alopecia, nausea, anemia and neutropenia, with grade 3 neutropenia frequently reported [12,15]. Cardiac safety remains an important concern, with asymptomatic declines in LVEF ranging from 5% in NeoSphere to 7%-16% in TRYPHAENA, and 2%-7.6% of patients showing decreases below 50% in GeparSepto [12,13,33]. Similarly, the NEOPERSUR study reported anti-HER2-related adverse effects in 11.9% of patients, including grade 3-4 cardiac events in 1.9% [6]. In comparison, our study observed cardiac toxicity in 10 patients, with one case requiring permanent discontinuation of therapy due to heart failure. These results are consistent with published data, confirming that while anti-HER2 therapy is generally well tolerated, cardiac toxicity remains the principal adverse event of concern.

This study has several limitations that should be considered when interpreting the results. First, its retrospective design may introduce selection and information biases. The relatively small sample size and the absence of a control group also limit the statistical power and reduce the generalizability of the findings. In addition, the RCB score, which provides a more detailed assessment of post-treatment response, was not available and therefore could not be incorporated into the analysis. Treatment heterogeneity represents another important limitation: although all patients received trastuzumab, only a minority were treated with dual HER2 blockade, restricting the ability to fully assess treatment effects across different anti-HER2 strategies. Finally, variations in chemotherapy regimens and follow-up duration may further influence the robustness of the conclusions. Larger, prospective studies using standardized response evaluation tools and uniform therapeutic protocols are needed to validate these findings and guide optimized management of HER2-positive breast cancer.

## Conclusions

In summary, our study reinforces the prognostic value of pCR in HER2-positive breast cancer, confirming its strong association with improved PFS and its enhancement through anti-HER2 therapies. Beyond therapeutic efficacy, our findings emphasize the interplay between treatment response and tumor-specific factors such as grade, nodal involvement, HR status, and proliferative index. These results underscore the need for a more integrated approach that combines clinical, pathological, and molecular determinants to refine prognostic stratification. Future research should not only rely on larger prospective cohorts but also explore novel biomarkers, genomic signatures, and immune-related parameters to better capture tumor heterogeneity. Ultimately, such efforts may pave the way toward more personalized therapeutic strategies, raising further questions on how best to individualize treatment intensity, optimize sequencing of systemic therapies, and identify patients who could benefit from treatment de-escalation versus escalation.
